# Patient-reported outcome metrics following total knee arthroplasty are influenced differently by patients’ body mass index

**DOI:** 10.1007/s00167-018-4853-2

**Published:** 2018-02-07

**Authors:** J. M. Giesinger, F. L. Loth, D. J. MacDonald, K. Giesinger, J. T. Patton, A. H. R. W. Simpson, C. R. Howie, David F. Hamilton

**Affiliations:** 1 Innsbruck Institute of Patient-centered Outcome Research (IIPCOR), Innsbruck, Austria; 2 University of Innsbruck, Innsbruck, Austria; 3 Department of Orthopaedics and Trauma, University of Edinburgh, Chancellors Building, 49 Little France Crescent, Edinburgh, EH16 4SB UK; 4 Department of Orthopaedics, Kantonsspital, St. Gallen, St Gallen Switzerland

**Keywords:** Total knee arthroplasty, Obesity, Patient-reported outcome, Forgotten joint score-12, Oxford Knee Score

## Abstract

**Purpose:**

This study investigated the impact of body mass index (BMI) on improvement in patient outcomes (pain, function, joint awareness, general health and satisfaction) following total knee arthroplasty (TKA).

**Methods:**

Data were obtained for primary TKAs performed at a single centre over a 12-month period. Data were collected pre-operatively and 12-month postoperatively with the Oxford Knee Score (OKS) measuring pain and function, the EQ-5D-3L measuring general health status, the Forgotten Joint Score-12 (FJS-12) measuring joint awareness and a single question on treatment satisfaction. Change in scores following surgery was compared across the BMI categories identified by the World Health Organization (< 25.0, 25.0–29.9, 30.0–34.9, 35.0–39.9 and ≥ 40.0). Differences in postoperative improvement between the BMI groups were analysed with an overall Kruskal–Wallis test, with post hoc pairwise comparisons between BMI groups with Mann–Whitney tests.

**Results:**

Of 402 patients [mean age 70.7 (SD 9.2); 55.2% women] 15.7% were normal weight (BMI < 25.0), 33.1% were overweight (BMI 25.0–29.9), 28.2% had class I obesity (BMI 30.0–34.9), 16.2% had class II obesity (BMI 35.0–39.9), and 7.0% had class III obesity (BMI ≥ 40.0). Postoperative change in OKS (n.s.) and EQ-5D-3L (n.s.) was not associated with BMI. Higher BMI group was associated with less improvement in FJS-12 scores (*p* = 0.010), reflecting a greater awareness of the operated joint during activity in the most obese patients. Treatment satisfaction was associated with BMI category (*p* = 0.029), with obese patients reporting less satisfaction.

**Conclusions:**

In TKA patients, outcome parameters are influenced differently by BMI. Our study showed a negative impact of BMI on postoperative improvement in joint awareness and satisfaction scores, but there was no influence on pain, function or general health scores. This information may be useful in terms of setting expectations expectation in obese patients planning to undergo TKA.

**Level of evidence:**

Level 1.

## Introduction

Total knee arthroplasty (TKA) is one of the most successful interventional procedures known to medicine, achieving improvements in patient health status comparable to coronary revascularisation and renal transplant [17]. Perhaps due to this success, operative rates have doubled in the last decade, with around 100,000 procedures now carried out annually in the UK alone [7]. Provision of such surgical volume is challenging and increasingly care commissioning bodies are looking for ways to ration services. A perceived poorer outcome for obese patients has recently led to the introduction of pre-operative weight thresholds for consideration of total knee arthroplasty in some parts of the United Kingdom. These thresholds have been introduced despite evidence that there is no reduction in interventional cost effectiveness when considering BMI [11].

Worldwide levels of obesity are rising rapidly; over 500 million people are currently classified as obese, with the emotive target of 1 billion individuals expected to be reached by 2030 [44]. A quarter of the population of developed countries are already reported as being obese presenting a major challenge for today’s health care systems [14]. Obesity is directly related to a number of conditions (such as coronary heart disease, hypertension and diabetes [24, 26]) and also to an increased incidence and progression of knee osteoarthritis. As such, obese patients are more likely to undergo total knee arthroplasty (TKA) at younger age than non-obese patients [16]. Postoperatively, obese TKA patients are considered to have higher risks of infection [36] and revision [1, 30, 43]; risks often compounded by the associated comorbidities frequently found in such patients [29]. Excessive body mass index (BMI) has been associated with low performance on objective outcome measures of physical function [15, 28], with impaired health-related quality of life [34] and with low treatment satisfaction [27, 35] also reported.

Results from other studies, however, present an inconsistent picture, with some suggesting no impact of BMI on postoperative recovery [5, 32, 37], and others a negative impact [33] or even a positive impact [8] of a high BMI. As such it is difficult to council obese patients as to their likely outcome pre-operatively. The differing results may partly be explained by variation of assessed outcome parameters and tools employed to assess this. A comprehensive evaluation of the impact of BMI on different outcome metrics that evaluate separate aspect on patient outcomes is lacking to help set patient expectations of surgery. The objective of this study was to investigate the impact of BMI on change in patient-reported pain, function, joint awareness, health status and satisfaction following total knee arthroplasty.

## Materials and methods

Patients undergoing primary total knee arthroplasty were assessed prospectively at a single NHS teaching hospital during 2014. The study centre is the only hospital receiving adult referrals for a predominantly urban regional population of around 850,000. Procedures were carried out by consultant orthopaedic surgeons and their supervised trainees using the Triathlon total knee replacement (Stryker) via a medial parapatellar approach. A measured resection technique was employed using cruciate retaining devices; routine postoperative protocols were followed in all cases. There were no special considerations made for patients based on their BMI. Data were collected with informed consent. Ethical approval for this study was obtained from the Scotland A Research Ethics Committee (11/AL/0079).

Paper questionnaires were employed for this study. Patients completed the forms at time of hospital pre-admission clinic, 2 weeks prior to surgery, and then via postal follow-up. Sociodemographic data and BMI score were collected pre-operatively. Patient-reported outcome questionnaires (Oxford knee Score, Forgotten Joint Score-12 and EQ-5D) were completed pre-operatively and at 12 months postoperatively. Patient satisfaction was assessed 12 months postoperatively.

## Patient-reported outcome questionnaires

### Forgotten Joint Score-12

The Forgotten Joint Score-12 (FJS-12; [6]) is a 12-item patient-reported outcome (PRO) measure of joint awareness in patients with knee or hip pathologies. The total score derived from the individual questions ranges from 0 to 100 with high scores indicating good outcome, i.e. a low level of joint awareness. The questionnaire has shown good reliability and validity in psychometric analyses [6, 19, 40, 41].

### Oxford knee score

The Oxford Knee Score (OKS; [13]) assesses pain and function in patients undergoing knee surgery. This widely used score has been shown to have good psychometric properties [12, 21]. The sum score derived from the items has a range from 0 to 48 points, with high scores indicating good outcome. An alternative scoring method calculating a separate pain and function score has been described by Harris et al. [20].

### EQ-5D-3L

The EQ-5D-3L [38] is a generic self-report questionnaire measuring the patient’s health status. The instrument consists of five questions covering self-care, mobility, depression/anxiety, pain and usual activities. From these five questions, a health utility can be calculated with a score of 1 reflecting full health, 0 indicating a health state equalling death and negative values describing health states that patients consider worse than being dead. This widely used questionnaire has shown satisfying measurement characteristics in knee patients [10].

### Treatment satisfaction

Satisfaction with TKA was assessed 1 year postoperatively using a single item question “How satisfied are you with your operated knee?” with five response categories (very satisfied, satisfied, unsure, dissatisfied, very dissatisfied). Patients were additionally asked whether they “would undergo the procedure again”? using the same 5 point response matrix.

### Statistical analysis

Sample characteristics are given as means, standard deviations, ranges, and frequencies. To assess the impact of BMI on postoperative improvement we categorised patients into five BMI groups following the categorization by the World Health Organization (WHO; [39]):

Normal weight: BMI < 25.0.

Overweight: BMI 25.0–29.9.

Class I obesity: BMI 30.0–34.9.

Class II obesity: BMI 35.0–39.9.

Class III obesity: BMI ≥ 40.0.

Power analysis was done for pairwise comparisons with non-parametric Mann–Whitney tests. A sample size of *N* = 60 per group provides 80% power (alpha = 0.05, two sided) to detect a difference with an effect size of Cohen’s *d* = 0.53. Comparing a group of *N* = 60 against a group of *N* = 30 and *N* = 120, respectively, allows to detect an effect size of *d* = 0.65 and *d* = 0.46. The group of underweight patients (BMI less than 18.5) was combined with the group of normal weight patients (18.5–24.99) due to the very low number of underweight patients. For each of the BMI groups we calculated the median and the 25th and 75th percentile as a measure of dispersion for the outcome scores at pre-surgery and 12 months as well as for the change between these two time points, i.e. postoperative improvement. Differences in postoperative improvement and in satisfaction at 1-year follow-up between the BMI groups were analysed with an overall Kruskal–Wallis test. In case of a statistically significant overall test, post hoc pairwise comparisons were conducted between BMI groups with Mann–Whitney tests. In addition, the association of BMI with outcome scores was analysed separately at pre-operatively and at 12 months using a Kruskal–Wallis test. *p* values below 0.05 were considered to be statistically significant. All statistical analyses were done in SPSS 24.0.

## Results

### Patient characteristics

Data from 402 TKA patients were analysed. Mean age was 70.7 (SD 9.2) years and 55.2% were women. Most patients were classified as being overweight (33.1%, BMI 25.0–29.9) or having class I obesity (28.1%; BMI 30.0–34.9). Twenty-eight patients (7.0%) had class III obesity (BMI ≥ 40.0). More detailed information is given in Table 1.

**Table 1:** Sociodemographic and clinical patient characteristics (*N* = 402)

	Mean (SD)	*N* (%)
Age	70.7 (9.2)	
Sex		
Women		222 (55.2)
Men		180 (44.8)
Side of implant		
Left		199 (49.9)
Right		200 (50.1)
Missing		3
BMI (%)		
≤ 24.99	Normal weight	63 (15.7)
25.00–29.99	Pre-obesity	133 (33.1)
30.00–34.99	Class I obesity	113 (28.1)
35.00–39.99	Class II obesity	65 (16.2)
≥ 40.00	Class III obesity	28 (7.0)

### Impact of BMI on pain and function

**Fig. 1:**
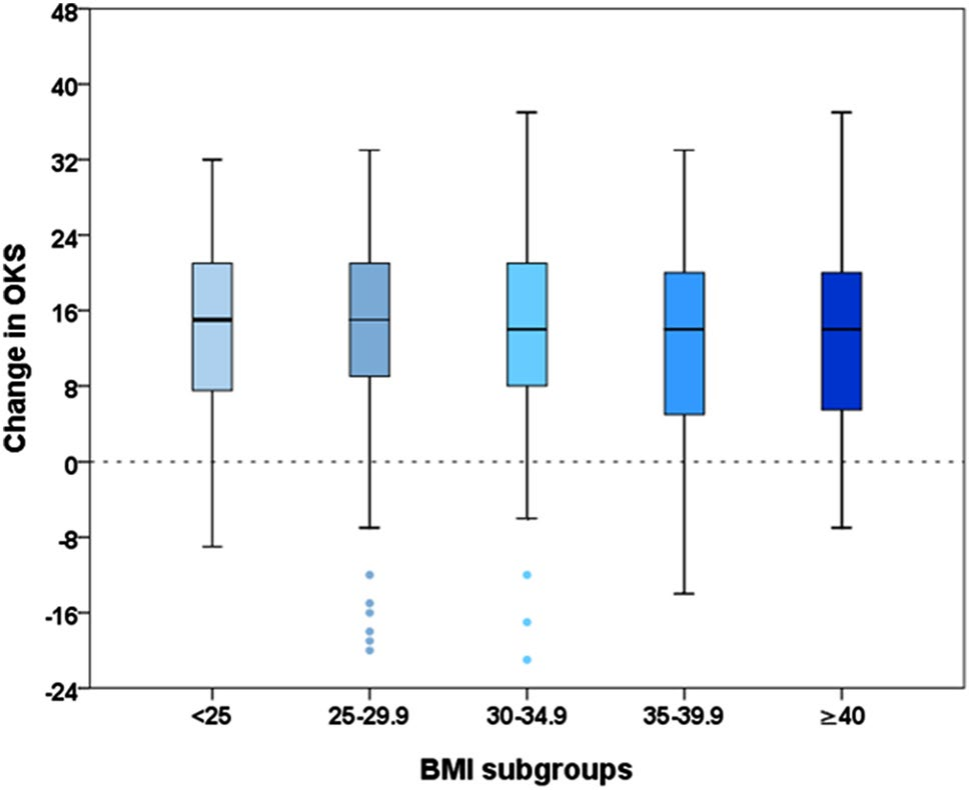
Comparison of postoperative change in OKS scores across BMI groups

OKS scores reflecting pain and function were associated with BMI pre-operatively (*p* < 0.001) and at 12-month follow-up (*p* < 0.001). Normal weight and overweight patients obtained highest scores pre-operatively (median OKS 21.0 and 22.0 points) and at 12-month follow-up (39.0 and 41.0 points), while patients with class III obesity showed lowest scores at both time points (15.5 and 27.5). Improvement of OKS scores from pre-surgery to 1-year follow-up did not differ significantly across BMI groups. Median improvement ranged from 14 (patients class I–III obesity) to 15 points (normal and pre-obese patients). For further details, see Table 2 and Fig. 1. Sub-analysis using separate scores for pain and function [20] confirmed no statistically significant association of postoperative improvement with the BMI groups.

**Table 2:** OKS scores for different BMI groups

BMI	OKS pre-surgery		OKS at 12 months		OKS improvement	
	Median	25th/75th percentile	Median	25th/75th percentile	Median	25th/75th percentile
< 25	21.0	17.0/27.0	39.0	26.0/44.0	15.0	7.0/21.0
25–30	22.0	17.0/28.5	41.0	28.0/45.0	15.0	8.5/21.5
30–35	19.0	15.0/25.5	36.0	27.0/41.5	14.0	8.0/21.5
35–40	19.0	15.0/24.0	34.0	25.5/41.5	14.0	5.0/20.0
≥ 40	15.5	10.3/19.8	27.5	22.0/38.5	14.0	5.3/20.0
*p* value	< 0.001		< 0.001		n.s	

### Impact of BMI on joint awareness

FJS-12 scores did not show a statistically significant association with BMI pre-operatively. Observed median scores were lowest in patients with class III obesity (4.2 points) and highest in patients with normal weight and class I obesity (both 8.3 points). At 12-month follow-up FJS-12 scores were significantly different across BMI groups (*p* = 0.002) with highest median scores found in normal weight patients (50.0 points) and pre-obese patients (56.3 points) and lowest scores in patients with class II obesity (33.3 points) and class III obesity (15.6 points). Median improvement rates, pre-operative to 12-month follow-up, were associated with BMI (*p* = 0.010) and ranged from 11.6 points (class III obesity patients) to 45.8 points (pre-obese patients).

**Fig. 2:**
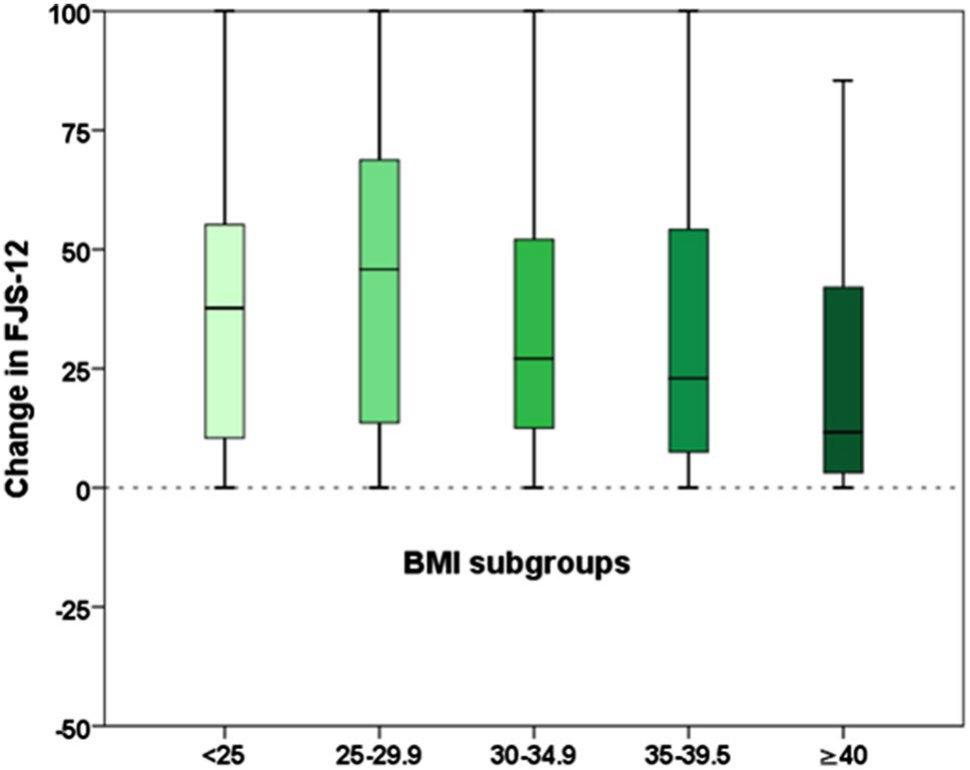
Comparison of postoperative change in FJS-12 scores across BMI groups

In pairwise comparisons, we found statistically significant differences between patients with class III obesity (postoperative change + 11.6 points) and normal weight (+ 37.7 points), pre-obese (+ 45.8 points) and class I obesity (+ 27.1 points) patients. In addition, class II obesity and pre-obese patients differed significantly (+ 22.9 vs + 45.8 points). Further details are given in Table 3 and Fig. 2.

**Table 3:** FJS-12 scores for different BMI groups

BMI	FJS-12 pre-surgery		FJS-12 at 12 months		FJS-12 improvement	
	Median	25th/75th percentile	Median	25th/75th percentile	Median	25th/75th percentile
< 25	8.3	2.8/16.7	50.0	18.8/75.0	37.7	10.4/56.3
25–30	7.5	2.1/16.7	56.3	20.8/81.3	45.8	13.1/68.8
30–35	8.3	1.6/16.7	35.4	22.6/63.5	27.1	11.5/53.1
35–40	6.3	2.3/14.6	33.3	17.7/63.5	22.9	6.9/54.2
≥ 40	4.2	0.0/6.3	15.6	7.2/52.6	11.6	2.6/44.3
*p* value	n.s		0.002		0.010	

Pairwise comparisons for BMI groups	*p* value
Normal weight vs pre-obesity	n.s
Normal weight vs class I obesity	n.s
Normal weight vs class II obesity	n.s
Normal weight vs class III obesity	0.027
Pre-obesity vs class I obesity	n.s
Pre-obesity vs class II obesity	0.013
Pre-obesity vs class III obesity	0.003
Class I obesity vs class II obesity	n.s
Class I obesity vs class III obesity	0.046
Class II obesity vs class III obesity	n.s

### Impact of BMI on general health

**Fig. 3:**
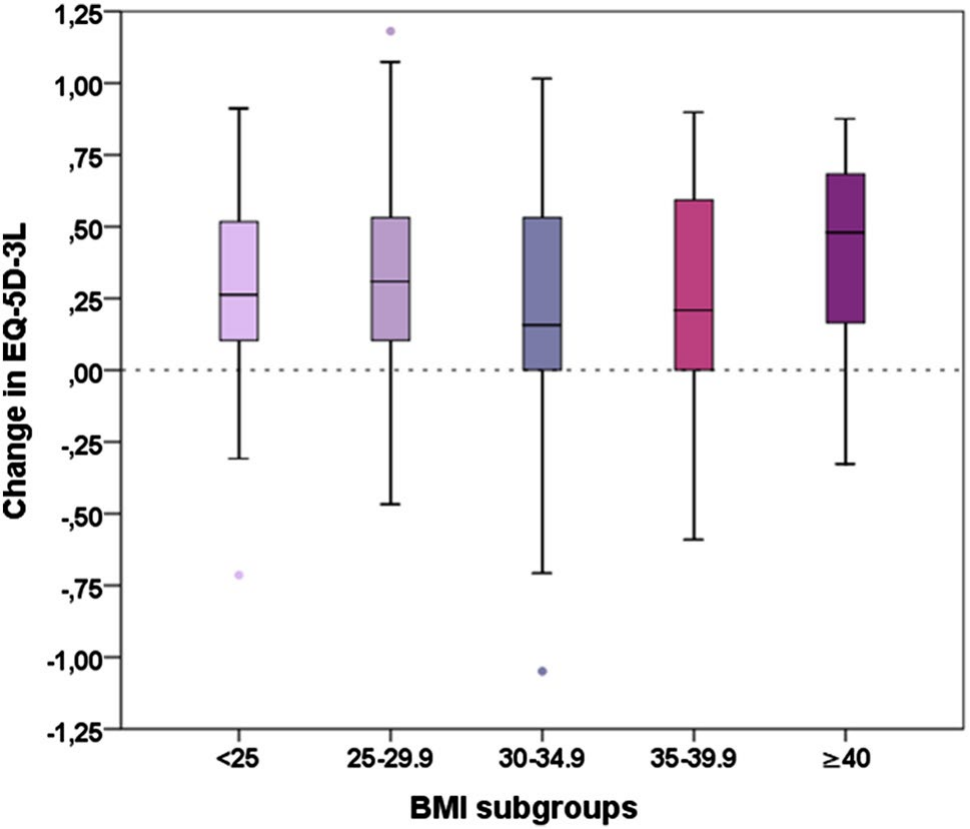
Comparison of postoperative change in EQ-5D-3L scores across BMI groups

General health measured with the EQ-5D-3L was associated with BMI pre-operatively (*p* = 0.001) and at 12-month follow-up (*p* < 0.001) with lower BMI being related to better general health. Pre-operatively, patients with BMI < 35 showed the same median score (0.62), whereas class II and III obesity patients showed substantially lower scores (0.36 and 0.16). At 12-month follow-up, patients with BMI > 30 scored 0.69 (median) whereas normal weight patients obtained a median score of 0.76 and pre-obese patients a median score of 0.80. Score change between pre-surgery and 12-month follow-up was not significantly different between the five BMI groups. Details are given in Table 4 and Fig. 3.

**Table 4:** EQ-5D-3L scores for different BMI groups

BMI	EQ-5D pre-surgery		EQ-5D at 12 months		EQ-5D improvement	
	Median	25th/75th percentile	Median	25th/75th percentile	Median	25th/75th percentile
< 25	0.62	0.16/0.69	0.76	0.69/1.00	0.26	0.10/0.53
25–30	0.62	0.16/0.69	0.80	0.69/1.00	0.31	0.09/0.53
30–35	0.62	0.19/0.69	0.69	0.55/0.81	0.16	0.00/0.53
35–40	0.36	0.10/0.69	0.69	0.59/0.80	0.21	0.00/0.60
≥ 40	0.16	0.01/0.44	0.69	0.52/0.80	0.48	0.16/0.69
*p* value	0.001		< 0.001		n.s	

### Impact of BMI on treatment satisfaction

**Fig. 4:**
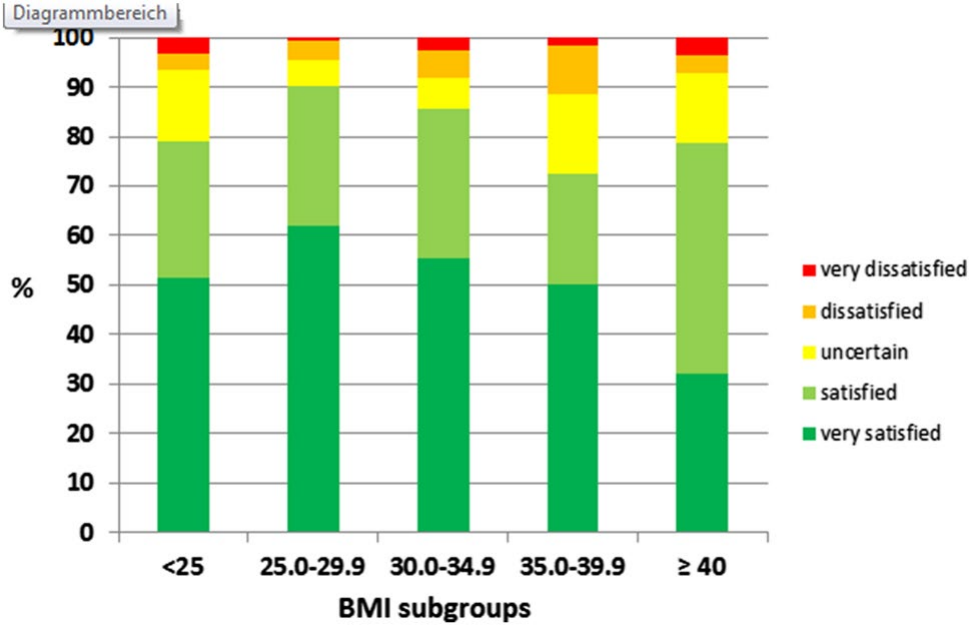
Comparison of postoperative satisfaction scores across BMI groups

BMI groups differed significantly in postoperative treatment satisfaction (*p* = 0.029). Percentage of very satisfied and satisfied patients across the five BMI groups was as follows: in normal weight patients 51.6% were “very satisfied” with a further 27.4% “satisfied”; in overweight patients these percentages were 62.1 and 28.0%; in class I obesity patients 55.5 and 30.0%; in class II obesity patients 50.0 and 22.6% and in class III obesity patients 32.1 and 46.4%, respectively. Pairwise comparisons showed a significant differences in postoperative satisfaction between overweight patients and class II or III obesity (*p* = 0.027 and *p* = 0.003) as well as between patients with class I obesity and those with class III obesity (*p* = 0.046), Fig. 4. There was no statistically significant association between BMI class and willingness to undergo the procedure again.

## Discussion

The prescient finding of this study was that patient BMI at the time of surgery influenced patient-reported outcome measures to different extents. Substantial differences between patients in the various BMI categories were observed for the OKS and the EQ-5D at pre-surgery as well as at 12-month follow-up. The association of FJS-12 scores with BMI was statistically significant at 12-month follow-up, but failed to reach statistical significance pre-surgery. Comparison of postoperative improvement across the BMI groups showed no statistically significant differences for the OKS and the EQ-5D-3L, whereas significant differences in postoperative improvement were observed for the FJS-12. For the FJS-12, the improvement for patients with class III obesity was significantly lower than for patients with normal weight, pre-obesity or class I obesity. Patients with class II obesity showed less improvement than pre-obese patients. This indicates that higher BMI is associated with being more aware of the joint following TKA. Similarly, higher BMI was also found to be associated with less treatment satisfaction.

The literature on the impact of BMI on pain and functional outcomes following TKA is somewhat conflicted. Amin et al. [2] suggest worse outcomes in patients with BMIs > 40 compared to patients with BMI < 30 in a case-matched study of patients, Hash et al. [23] report no difference comparing patients with BMI < 30 and BMI > 35, and Chen et al. [8] suggest greater improvement in the more obese patient groups. In line with our results, Baker et al. [5] reported no differences in OKS improvement comparing patients in class I, II and III obesity, and Ayyar et al. [4] report the same postoperative gain in OKS dichotomising patients by BMI above or below 30.

Rates of postoperative satisfaction were lower for patients in the most overweight categories, with those in class II and III obesity reporting lower satisfaction than patients in the ‘overweight’ category. Satisfaction scores following TKA have been demonstrated to be influenced by the experience of healthcare delivery and meeting pre-operative expectations to the same extent as they are by achieving symptomatic pain relief [18]. As such the statistical variation in satisfaction scores in the most obese categories may be related to context-specific issues in care delivery for obese patients or in pre-operative expectation management, as pain score change was equivalent between BMI groups.

Strengths of this study include the prospective design, and availability of data from joint-specific as well as generic outcome measures that allowed to demonstrate the differential impact of BMI on outcome after TKA and a sample size that allowed to compare BMI subgroups as suggested by the WHO. This is also the first evaluation of joint awareness measured with the FJS-12 investigating the impact of BMI on postoperative recovery. A limitation is that we recorded the patients’ BMI pre-operatively and could not account for any weight changes that may have taken place by the 12-month postoperative review. However, current literature indicates that most patients maintain their weight after TKA [3] suggesting a minor impact of weight change on our results. Two-thirds of patients in this cohort clustered in the overweight/pre-obese and class I obese categories. This is consistent with the suggested rise in BMI in the developed nations; however, may be specific to the UK. We do not know how these demographic data reflect wider European values; however, it is important to point out that any variation should not be expected to impact the outcome score associations reported but rather the prevalence of obesity parameters internationally. Obesity levels may be associated with wider socioeconomic parameters that we did not evaluate this specifically in our study. This can be considered as a limitation; however, again, it is unlikely to impact the relationships between outcome scores and BMI, which was the purpose of this evaluation. The epidemiological study design limits our ability to comment on any technical differences relating to the index procedure that may be associated with differing outcome scores. Various factors, such as implant design [9, 45], surgical philosophy [22] and implant positioning considerations [31, 42] have all been suggested to influence patient outcomes. The consistency of implant and surgical technique utilised in this cohort mitigate any such issues. Though causal associations between implant position factors and outcome scores cannot be established with data such as these, any tendency of obesity to cause predictable variation in implant positioning would be reflected in our data. That no differences were apparent in pain or function scores in our cohort suggests this is not a notable concern.

This study highlights the clinical benefit of total knee arthroplasty irrespective of obesity class when pre-operative disability is taken into account. Those with higher BMI report increased pain and dysfunction pre-operatively, perhaps reflecting significant case mix selection in the referral process. Complication risks are greater in the most obese patients; however, even in the “high risk” patients, TKA remains a cost-effective intervention [25]. As such, the efficacy of applying BMI thresholds to determine access to total knee arthroplasty is questionable. The information provided by this study may be useful in the pre-operative setting counseling obese patients as to the likely clinical outcomes of the joint arthroplasty.

## Conclusion

This study adds to a growing body of literature on the impact of BMI on postoperative improvement in TKA patients. The findings suggest that benefit of TKA in terms of pain, function and general health is not related to pre-operative BMI. More discerning outcome metrics, however, like being able to forget about the artificial joint in everyday life, may be more sensitive to BMI as obese patients seem to experience less gain. Perhaps most interesting obese patients are less satisfied with outcomes. Future research should account for the finding that benefit from TKA may differ for obese patients across the various outcome domains commonly measured in such patients, and evaluate the differences found in higher level metrics.
